# Microstructure and Physicochemical Properties of Light Ice Cream: Effects of Extruded Microparticulated Whey Proteins and Process Design

**DOI:** 10.3390/foods10061433

**Published:** 2021-06-21

**Authors:** M Kamal Hossain, Miroslav Petrov, Oliver Hensel, Mamadou Diakité

**Affiliations:** 1 Department of Agricultural and Biosystem Engineering, Faculty of Organic Agricultural Sciences, University of Kassel, Nordbahnhofstr. 1a, 37213 Witzenhausen, Germany; ohensel@uni-kassel.de; 2 Department of Animal-Derived Food Technology, Faculty of Food Technology, Fulda University of Applied Sciences, Leipziger Straße 123, 36037 Fulda, Germany; petrovv92@gmail.com (M.P.); mamadou.diakite@lt.hs-fulda.de (M.D.)

**Keywords:** extruded microparticulation, hot extrusion, light ice cream, pasteurization, homogenization, process techniques

## Abstract

This study aimed to understand the influence of extruded microparticulated whey proteins (eMWPs) and process design in light ice cream processing by evaluating the microstructure and physicochemical properties. The inulin (T1), a commercial microparticulated whey protein (MWP) called simplesse (T2), a combination (T3), as well as eMWPs (as 50% volume of total particles): d_50_ < 3 µm (T4), and d_50_ > 5 µm (T5) were used as fat replacers. The first process design was pasteurization with subsequent homogenization (PH). The second process was homogenization with subsequent pasteurization (HP) for the production of ice cream (control, 12% fat, *w*/*w*; T1 to T5, 6% fat, *w*/*w*). The overrun of light ice cream treatments of PH was around 50%, except for T4 (61.82%), which was significantly higher (*p* < 0.01). On the other hand, the overrun of HP was around 40% for all treatments except T1. In both the PH and HP groups, the color intensities of treatments were statistically significant (*p* < 0.001). The melting behavior of light ice cream was also significantly different. The viscosity of all treatments was significant (*p* < 0.05) at a shear rate of 64.54 (1/s) for both cases of process design. A similar firmness in both the PH and HP groups was observed; however, the products with eMWPs were firmer compared to other light ice creams.

## 1. Introduction

Extruded microparticulated whey proteins (eMWPs) produced through a thermomechanical process with a high concentration of milk proteins [1,2,3] are able to enhance the physicochemical properties of dairy products such as ice cream [4] and yogurt [5]. Microparticulated whey proteins (MWPs) are commercially available in powder form and are commonly known as fat replacers [6]. Protein-based fat replacers are usually derived from whey protein concentrates (WPCs), which simulate fat globules and enhance the sensory quality of frozen dairy products, especially creaminess [5]. MWPs are incorporated into light dairy products, especially ice cream, to improve their microstructural properties and creaminess. The size of microparticles (0.1–10 µm) is an important determinant for the sensory attributes of light dairy products [7] and physicochemical properties, such as firmness, viscosity, and melting behavior [4,5,8].

Ice cream is a frozen complex food matrix consisting of ice crystals, fat globules, and air bubbles dispersed in an unfrozen viscous serum phase [4,9,10]. Typically, standard ice cream contains 10–12% fat [11], and light ice cream contains 5–7% fat [7]. The fat content and structure of ice cream affect its physicochemical properties. For example, adding milk fat to ice cream lowers the ice phase volume and stabilizes air bubbles, which are sensory qualities that are expected in ice cream [12,13,14]. In addition, these qualities affect the texture and color development of ice cream [15]. Higher consumption of fat increases the risk of obesity, diabetes, and coronary heart disease [4,6]. However, fat reduction in dairy products remains a challenge for the industry. Fat reduction affects the quality of dairy products, including light ice cream, such as deficiency in the flavor profile, disruption of the fat globule network, poor texture, and lower quality. As health-conscious consumers’ demand for light and non-fat ice cream is increasing, the quality of light ice cream products should not be compromised. Therefore, various protein- and carbohydrate-based fat replacers have been used to mitigate defects and enhance the quality of light ice cream [7,16].

Inulin improves the stability of foams and emulsions in ice cream. Several authors have suggested that carbohydrate-based fat replacers, including inulin, cellulose derivatives, and modified starches, can be used as texture modifiers and free water binders in a food matrix [6,17,18]. The use of inulin influences the physicochemical properties of light ice cream, resulting in increased viscosity, firmness, and air incorporation as a result of the high degree of polymerization [12,16] as well as a decreased meltdown rate [7].

The formulation of ice cream, process design, and effects of different microstructural elements, such as air cells, ice crystals, and fat globule clusters, are responsible for the properties of ice cream [9,19]. Several studies have shown that high shear force and microfluidization of fat replacers affect the physical and structural properties of frozen dairy products [19,20]. A few recent studies have also shown that homogenization at high pressure (200–400 MPa) decreases the need for stabilizers [21] and facilitates greater interaction between proteins and polysaccharides, thus leading to a stable network structure [22]. In addition, the temperature has a considerable influence on the crystallization process, which determines the microstructural properties of ice cream [22]. These properties of light and low-fat ice cream are also significantly affected by the use of stabilizer–emulsifier hydrocolloids, especially xanthan gum [4]. Although several studies have investigated the stabilization process of ice cream using hydrocolloids, crystallization, and microstructures of low-fat ice cream, to date, several studies have been done on the process design of ice cream production (e.g., pressure and temperature), the application of commercial MWPs, and the microstructure of light ice cream. In addition, the rehydration effects of powder-based MWPs on the physicochemical and microstructural properties of light ice cream have not been fully explored.

Therefore, this study aimed to investigate the effects of eMWPs, which are hydrated, on the microstructure and physicochemical properties of light ice cream. It also considers the effects of different microparticle sizes (d_50_ < 3 µm and d_50_ > 5 µm) and process designs (pasteurization with subsequent homogenization (PH) and homogenization with subsequent pasteurization (HP)) on light ice cream (6% fat, *w*/*w*) compared to a control sample (12% fat, *w*/*w*) and powder-based MWPs.

## 2. Materials and Methods

### 2.1. Raw Materials

The raw materials used in the study, which were collected from various sources, are listed in Table 1. Pasteurized liquid skimmed milk (Hochwald Foods GmbH, Hünfeld, Germany), skimmed milk powder (Friesland Campina GmbH, Heilbronn, Germany), whey protein concentrates (Sachsenmilch Leppersdorf GmbH, Wachau, Germany), and pasteurized cream (Privatmolkerei Naarmann GmbH, Neuenkirchen, Germany) were used. A commercially available stabilizer–emulsifier blend, Palsgaard^®^ (Palsgaard A/S, Juelsminde, Denmark) and inulin (Nu U Nutrition Limited, Manchester, UK), was used for ice cream production. Commercially available microparticulated whey proteins (Simplesse^®^ 100) were kindly provided by CP-Kleco GmbH (Großenbrode, Germany).

### 2.2. Experimental Design

**Figure 1:**
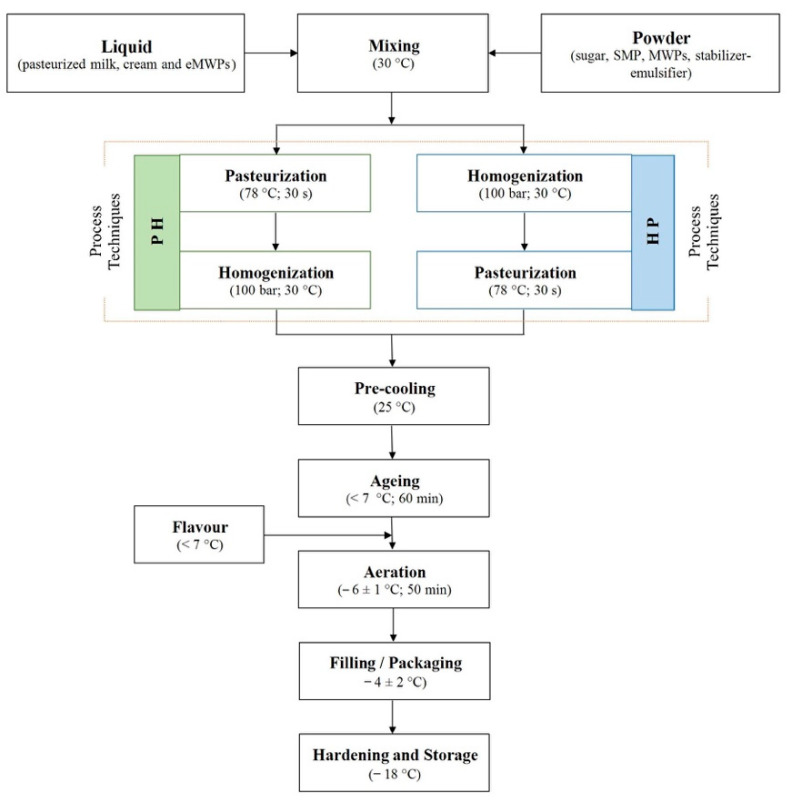
Flow chart of ice cream manufacturing with consideration of process design.

All light ice cream (6% fat, *w*/*w*) treatments (T1–T5) with and without microparticulated whey proteins and the control ice cream sample (12% fat, *w*/*w*) were prepared in the laboratory (Table 1). Both the PH and HP process designs were applied in each experiment (Figure 1). The total number of experiments (*n* = 12) was equal to the number of treatments plus the control, multiplied by the number of process designs.

A full factorial experiment design was chosen, and the experiments were conducted in triplicate. As the eMWPs are hydrated, to adjust the dry matter and other elements, such as lactose, of the ice cream mix, SMP and a higher number of eMWPs were used, although the C_protein_ and dry matter (DM) in light ice cream treatments were kept similar. These are key factors for the freezing point of the ice cream mix. The microstructure and physicochemical properties of ice cream treatments, such as chemical composition (fat, protein, dry matter), overrun, firmness, melting behavior, viscosity, and color properties, were determined with the following procedures.

### 2.3. eMWPs and Particle Size Distribution

The eMWPs were produced with a co-rotating twin-screw extruder (Leistritz Extrusionstechnik GmbH, Nürnberg, Germany), which had an L/D ratio (screw size) of 40. The microparticulation process and particle size distribution were performed and assessed according to the process explained by Hossain et al. [2].

### 2.4. Manufacturing of Ice Cream

Vanilla ice cream samples were formulated with 6% (light) and 12% (control) milk fat, and MWPs were added to replace the fat in the light ice cream treatments [7,23]. The dry matter of light ice cream was kept constant throughout the treatments (Table 1), but the content of liquid skimmed milk, cream, and MWPs was varied to account for the dry matter and protein concentration of ice cream mixes. All ingredients were weighed separately and gently mixed using a hand beater at room temperature for complete dispersion of the solids. A 1000 g batch was produced for each treatment. The mixes were heated in a 30 °C water bath to achieve appropriate dispersion. The control sample was prepared with the process explained in the literature [7]. The mixes were pasteurized at 78 °C for 30 s and then homogenized (APV Systems, Albertsund, Denmark) at room temperature with a pressure of 100 bar in a single-stage process. Different process designs such as, first, pasteurization and subsequent homogenization (PH) and, second, homogenization and subsequent pasteurization (HP) were considered to investigate the effects of fat replacers on the physicochemical and microstructural quality of ice cream (Figure 1). Then, the mixes were cooled at <7 °C and stored in the refrigerator for 1 h of aging. After aging, vanilla flavor was added to the mixes. All ice cream samples were made using the Gelato 3000 ice cream maker (Nemox International, Brescia, Italy). Before the mix was added, the ice cream maker was pre-cooled for 10 min to reduce the temperature difference between the mix and the contact surface of the ice cream maker. This was done to avoid the formation of large crystals. Roughly 50 mL of 40% (*v*/*v*) alcohol (propanol) solution was added between the inner and outer bowls of the ice cream maker for fast and consistent cooling. The ice cream was ready after 50 min of aeration. Overrun was measured, and plastic sample containers were filled with the ice cream. The aeration temperature (−6 ± 1 °C) was recorded right before measuring the overrun of the air-incorporated ice cream. The samples were transferred to the freezer at −18 °C for hardening and were stored until the analytical experiments were performed.

### 2.5. Chemical Composition Analysis

The composition of ice cream samples was measured using a near-infrared (NIR) spectrometer, NIRFlex N-500 (BÜCHI Labortechnik GmbH, Essen, Germany). The parameters and methods of NIR analysis, explained in the literature [5,24], were used with slight modification. Measurements were taken at an ambient temperature of 20 ± 2 °C and a wavelength range of 400–1000 nm. The spectrum of each sample was considered to be the average of 48 successive scans of the samples. The chemical composition (protein, fat, and dry matter) and spectra of ice cream samples were recorded.

### 2.6. Melting Behavior

The melting behavior of the ice cream samples was evaluated after storage at −18 °C for eight days. For evaluation, 35 g of ice cream was placed on a 1 mm mesh stainless steel sieve fitted into a funnel, and the melted ice cream drained into a transparent glass cylinder at a room temperature of 20 ± 1 °C [6]. The time it took from the start of the experiment until the first drop of melted ice cream fell was recorded [25], and the weight of the melted ice cream in the cylinder was recorded every 20 min for 120 min [7]. The melting behavior was measured as the weight of melted ice cream versus time.

### 2.7. Overrun

The air incorporated into ice cream samples was calculated using a weight-based formula explained in the literature [7]. Overrun was measured using a 250 mL glass jar. The weights of the ice cream mix and air-incorporated ice cream were measured separately. Overrun was calculated as follows: overrun % = ((weight of mix − weight of same volume of ice cream)/weight of same volume of ice cream) × 100. The density of the milk constituents (fat = 0.93 g/mL, milk solid non-fat MSNF = 1.58 g/mL, and water = 1 g/mL) was considered to calculate the actual overrun of ice cream.

### 2.8. Rheological Properties of Ice Cream

#### 2.8.1. Texture analysis

Texture measurements were taken at product and room temperatures of 10 ± 1 °C and 20 ± 1 °C using a Stable Micro Systems texture analyzer, TA.XT*plus* (Texture Tech. Corp., NY, USA), equipped with an acrylic crystal cylindrical probe (ø = 25 mm). Ice cream samples were carefully positioned in the texture analyzer within a plastic sample cup (90 mm diameter × 60 mm height) filled to approximately 50 mm. The samples were stored at −18 °C for 14 days, and then, texture analysis was carried out using the method described by Akalin et al. [6] with slight modification. The parameters were as follows: speed before test, 3 mm/s; test speed, 3.3 mm/s; reverse speed, 3.3 mm/s; penetration distance, 15 mm; and trigger force, 0.049 N. Firmness was measured as the peak compression force (N) during penetration of the sample, and adhesiveness was measured as the reverse peak force (N) during withdrawal from the ice cream sample.

#### 2.8.2. Dynamic Viscosity Analysis

The viscosity of ice cream samples was measured with a rotary viscometer using the method explained by Hossain et al. [5] with some alterations in the sample preparation. The samples were taken from the freezer and left at room temperature (22 ± 2 °C) for 24 h before measuring the viscosity. As explained in the literature [26], the human chewing intensity (shear rate) ranges from 1.5 to 121.5 (1/s). This range was taken into consideration during viscosity measurements. Shear rate (γ), shear stress (τ), and dynamic viscosity (*η*) were derived from the measured values and factors specific to the measuring module [27].

### 2.9. Microstructure Analysis of Ice Cream

Microstructural images of the ice cream samples were created by scanning electron microscopy (SEM) at an acceleration voltage of 10 kV and high beam current (CamScan MV2300, EO Elektronen-Optik-Service GmbH, Dortmund, Germany). Frozen samples were prepared for microstructure observation with a rotational vacuum freeze-dryer, ALPHA 2–4 LSCplus (Martin Christ Gefriertrocknungsanlagen GmbH, Osterode am Harz, Germany), at temperatures lower than the glass transmission temperature to protect the actual structure of the ice cream [28]. Then, the samples were prepared for SEM by polishing, and they were sputter-coated with a thin layer of gold. Measurements were taken with a secondary electron (SE) detection system, and photographs were taken at various levels of magnification [29].

### 2.10. Color Properties

Ice cream color was measured using a d/8° portable spectral colorimeter (Hach Lange GmbH, Düsseldorf, Germany). The measurements were recorded with the CIELAB (International Commission on Illumination L*a*b*) color system using the method explained in the literature [6,13], where L* refers to brightness, a* refers to redness, and b* refers to yellowness. The total color difference (∆*E*) was calculated using the following equation [30]:

$$∆E=\sqrt{\left(L^{*}t_{x}-L^{*}c\right)2+\left(a^{*}t_{x}-a^{*}c\right)2+\left(b^{*}t_{x}-b^{*}c\right)2}$$

where *t_x_ = t*_1,2,3,4,5_ (i.e., the treatments) and *c* refers to the control sample. After each measurement, the contact surface of the colorimeter was rinsed with water and dried before taking the next measurement.

### 2.11. Statistical Analysis

Statistical analysis and data organization were performed using R-Core (2018, R-Studio, Boston, MA, USA) and XLSTAT (version 2019.3.1, Addinsoft, New York, NY, USA). For analysis, the treatments and control sample were measured in triplicate. One-way analysis of variance (ANOVA) was performed with statistical significance level set at *p* < 0.05 for all treatments. Pairwise comparisons of overrun, melting behavior, firmness, and viscosity were performed for all treatments subjected to both process designs by Tukey’s HSD (honest significant difference) test. Correlation and regression analyses were performed to assess the chemical composition and physicochemical properties of all treatments.

## 3. Results and Discussion

### 3.1. Effect of Fat Replacers on the Physicochemical Properties of Ice Cream

#### 3.1.1. Chemical Composition of Ice Cream Mixes

The analytical results for the major components of the ice cream mixes for all treatments and the control sample are described in Table 2. The fat, protein, and dry matter significantly differed (*p* < 0.05) within the group of process design (PH and HP). The dry matter of light ice cream was significantly similar in all treatments; only the control sample was different (Table 1). The fat content of ice cream mixes ranged from 6.05 ± 0.57 to 7.09 ± 0.30%; however, the control mixes had a fat content of 12.56 ± 0.77 to 13.04 ± 0.76%. For both process designs, the protein contents of T2, T4, and T5, which were made with MWPs and eMWPs, were significantly higher compared to the other treatments. Traditionally, ice cream has a protein content of 2.5–4.0% [7]. The protein content of ice cream mixes was significantly lower (*p* < 0.05) for T1 and T3 subjected to PH 5.07 ± 0.11% and 5.75 ± 46%, respectively, compared to all other treatments. Protein and fat aid in the development of ice cream’s overall structure, stability, and smoothness during emulsification and whipping [14]. The protein content had a strong, positive correlation with melting behavior (r = 0.74, *p* < 0.05) and a negative correlation with viscosity (r = −0.63, *p* < 0.05), as shown in Table S1. A reason for this stronger gel network in the ice cream matrix is because of the higher protein content [5]. Total dry matter contents of the control sample for PH and HP were 35.57 ± 1.59% and 35.91 ± 1.22%, respectively, and all other treatments ranged from 30.57 ± 1.97 to 32.91 ± 2.46% (Table 2). The dry matter has a major influence on the structural behavior of ice cream, with less dry matter resulting in larger ice crystals [31]. Although there is no precise standard for categorizing ice cream on the basis of its protein content, ice cream with a protein content of 5 g per serving (64.8 g) at 50% overrun can be considered to be super premium ice cream [7].

#### 3.1.2. Melting Behavior and Time until First Drop

**Figure 2:**
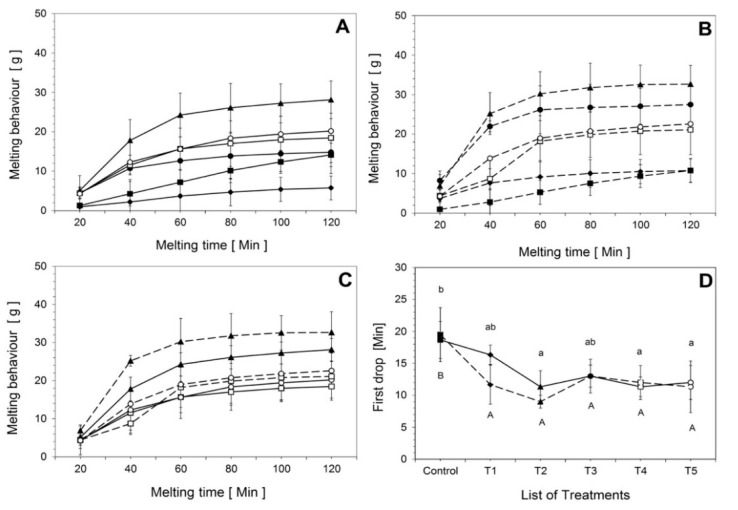
Melting behavior of ice cream treatments: Control (■), T1 (◆), T2 (▲), T3 (●), T4 (☐), and F5 (◯) for both process designs. Relationship between time and melting behavior: (**A**) solid line (—) represents PH, (**B**) dotted line (- - -) represents HP, (**C**) treatments with only MWPs and eMWPs, and (**D**) time until the first drop of melted ice cream at a room temperature of 20 ± 2 °C. The product temperature started from −15 ± 2 °C. Different letters (a,b) for PH and (A,B) for HP on top of the bars indicate significant differences at *p* < 0.05. The same letter indicates that the products are not significantly different from each other. Error bars indicate the standard deviation. See Table 1 for the definitions of treatment abbreviations.

The time until the first drop and melting behavior of ice cream treatments subjected to the two process designs are shown in Figure 2. There were notable differences between the time until the first drop for T1 and T2 (Figure 2D), and the melting behavior of T5 was significantly higher than that for all other treatments for both PH and HP (*p* < 0.05). For both process designs, the melting behavior of the control sample and T1 with inulin was significantly lower (Figure 2A,B). A similar result was found by Akalin et al. [6]. In general, there is a relationship between melting characteristics and thermal diffusivity. The existence of a higher fat content in ice cream reduces the thermal diffusivity, preventing heat transformation, which results in a lower melting rate [6,17]. It has been shown that the overall melting behavior is affected in light ice cream, which could be due to the reduced fat content [4]. The consistency of ice cream mixes has also been found to affect the melting properties [32]. Indeed, the treatment containing inulin exhibited less melting than the treatments with MWPs. The formation of a stable network and the melting behavior depend on the fat, protein, and hydrocolloid content used to produce light ice cream [4,7]. Protein-based fat alternatives provide better melting properties compared to carbohydrate-based alternatives [7]. However, the rehydration time of protein-based fat replacers can affect the melting behavior of ice cream, as shown in Figure 2C. The melting rate was lower for ice creams made with eMWPs compared to those made with powder-based MWPs for both process designs. The contributions of native proteins, the colloidal character of the proteins, and the rehydration time required to build protein networks were analyzed for microparticulated proteins. Similar conclusions were made by Liou et al. [33]. There were no differences in melting between ice cream made with PH and HP.

#### 3.1.3. Color of Ice Cream

Table 3 presents the color properties of all treatments and the control sample for both process designs (PH and HP). There were significant differences (*p* < 0.05) in the L*, a*, and b* values within the group of samples subjected to each process design. For both process designs, the brightness of the control sample was higher than that of the other treatments. However, the treatments did not show significant differences between PH and HP. T2, T3, T4, and T5, which were made with MWPs and eMWPs, were significantly different (*p* < 0.05) from the control, while T1 exhibited a similar behavior to the control sample. In general, the brightness (L*) of the ice cream increased significantly with increased fat content [30]. However, the a* values showed the opposite results, as also reported by Güven et al. [15]. The yellowness (b*) of T1 and T3 was significantly (*p* < 0.05) lower than that of other ice cream treatments, although the fat contents of T2, T4, and T5 were similar, and the b* value directly corresponded to the fat content of ice cream [6]. The total color differences of all treatments were calculated, and color was found to be unaffected by the presence of MWPs and eMWPs, even in the process design.

### 3.2. Rheological Properties

#### 3.2.1. Firmness and Incorporated Air in Ice Cream

**Figure 3:**
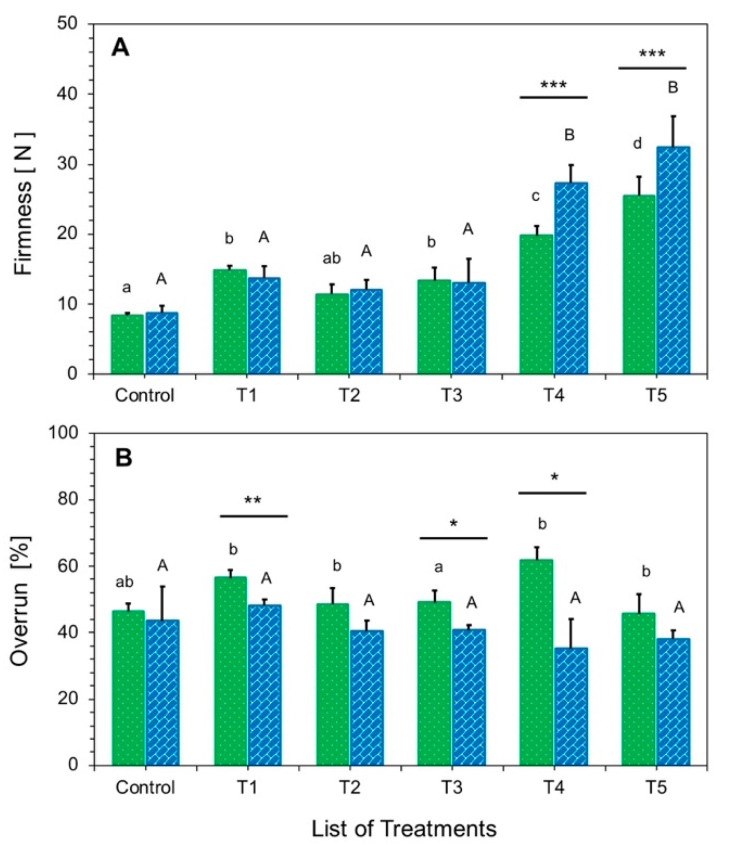
The firmness (**A**) and overrun (**B**) of ice cream treatments with two different process designs of PH and HP symbolized by green (■) and blue (■) bars, respectively. Different letters (a–d) for PH and (A, B) for HP on top of the bars indicate significant differences at *p* < 0.05. The same letter indicates that the products are not significantly different from each other. Error bars indicate standard deviation. See Table 1 for the definition of treatment abbreviations. The significance levels (* *p* < 0.05; ** *p* < 0.01; *** *p* < 0.001) between process designs.

The firmness of ice cream products is usually affected by several factors: total dry matter, viscosity, the volume of incorporated air, and the type and amount of stabilizer [4,6]. The significance (*p* < 0.05) of firmness and overrun is shown in Figure 3. The firmness of the control sample was markedly lower than that of other treatments because of the inverse relation of firmness to fat content (Table S1). Our observations were consistently similar to those of [34,35]. Treatments T1 and T3, which were made with inulin, had higher firmness because of the high degree of polymerization and the formation of microcrystals through dissolution in milk. These microcrystals interact in the ice cream matrix and increase firmness [16]. Higher firmness was also observed in T2, T4, and T5, which contained MWPs and eMWPs, although the dry matter contents of all respective treatments were similar to each other (Table 2). The firmness of T4 and T5 was high and statistically different (*p* < 0.001) between PH and HP; the firmness of treatments made with HP was markedly higher. The higher firmness may be due to the heat treatment of ice cream mixes after homogenization, which did not allow the breakdown of larger protein networks built by ß-lactoglobulin with a combination of stabilizer/emulsifier. Similar findings were reported in the literature [4,6].

The overrun of ice cream is greatly influenced by freezing and aeration temperature. Freezing that occurs too slowly results in the formation of larger ice crystals, whereas freezing that occurs too rapidly reduces the time for which air is incorporated into the ice cream. Overrun was statistically significantly higher (*p* < 0.05) for PH than for HP, as shown in Figure 3. In addition, higher overrun was observed in light ice cream containing inulin (T1). Lower overrun was observed in the light ice cream with eMWPs, except for T4, which could be due to heating failure during the production process. These results are supported by the work of [6]. A strong relation between overrun and firmness was observed (Table S1); T4 and T5 had lower overrun and, thus, were higher in firmness. A similar relation was described by Muse et al. [32]. In the case of overrun, statistical differences between PH and HP were detected for specific light ice cream treatments (T1, T3, and T4). The air incorporation in ice cream is influenced by total solids, fat content, and particle size distribution [12,36]. Air is a good insulator; therefore, ice cream with higher air incorporation tends to melt slower [9].

#### 3.2.2. Viscosity of Ice Cream

**Figure 4:**
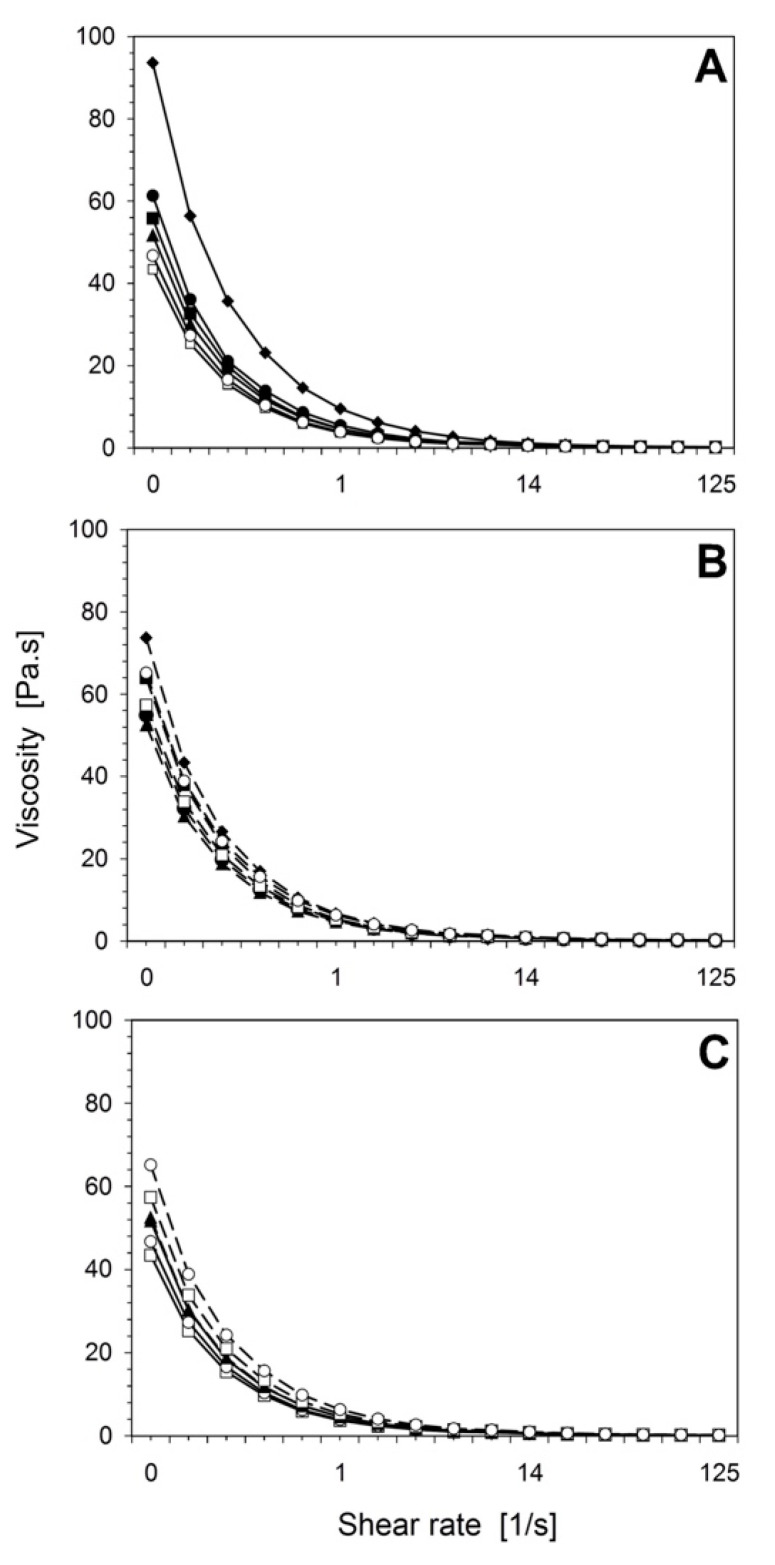
The viscosity of ice cream treatments: Control (■), T1 (◆), T2 (▲), T3 (●), T4 (☐), and F5 (◯) for both process designs. Relationship between viscosity and shear rate: (**A**) solid line (—) represents PH, (**B**) dotted line (- - -) represents HP, and (**C**) treatments with only MWPs and eMWPs at a room temperature of 20 ± 2 °C. The product temperature started from 5 ± 2 °C. See Table 1 for the definitions of treatment abbreviations.

Figure 4 shows the relationship between dynamic viscosity and the range of shear rates for the light ice cream samples made with PH and HP as well as various fat replacers. For both process designs, all the treatments and the control sample showed a negative relationship between shear rate and viscosity as well as shear-thinning behavior. This could be due to the structural breakdown of the ice cream matrix [37,38]. T1, which contained inulin, showed the highest viscosity (*p* < 0.05) compared to the other treatments (Figure 4A). Similar findings were reported by Yan et al. [4].

In general, the viscosity of the ice cream mix was influenced by the concentrations of stabilizer–emulsifier, protein, fat, MSNF, and total dry matter. Typically, a higher lactose content in the ice cream mix leads to a reduced freezing point, which increases the likelihood of lactose crystallization and, in turn, results in higher viscosity [4]. In this study, stabilizer–emulsifier was used in combination with xanthan gum to inhibit lactose crystallization and enhance the viscosity and mouthfeel. The functional properties of ß-lactoglobulin and α-lactalbumin facilitate gelation, thickening, and the formation of large protein networks after a specific heat-induced process. Heat treatment and the particle sizes (d_50_ > 5 µm) of MWPs resulted in higher viscosity for ice cream mixes subjected to HP compared to those subjected to PH, as shown in Figure 4C. The viscosities of T2, T4, and T5, which contained Simplesse and eMWPs with particle sizes of d_50_ < 3 µm and d_50_ > 5 µm, respectively, were significantly different (*p* < 0.01). The viscosity for PH had a strong negative correlation with firmness (r = −0.575, *p* < 0.05) and melting behavior (r = −0.684, *p* < 0.05). HP showed a similar relation as overrun (HP, r = −0.497, *p* < 0.05), shown in Table S1. Furthermore, there was a clear difference in the viscosities of T4 and T5 between the process designs.

### 3.3. Effect of Fat Replacers on Microstructural Properties

**Figure 5:**
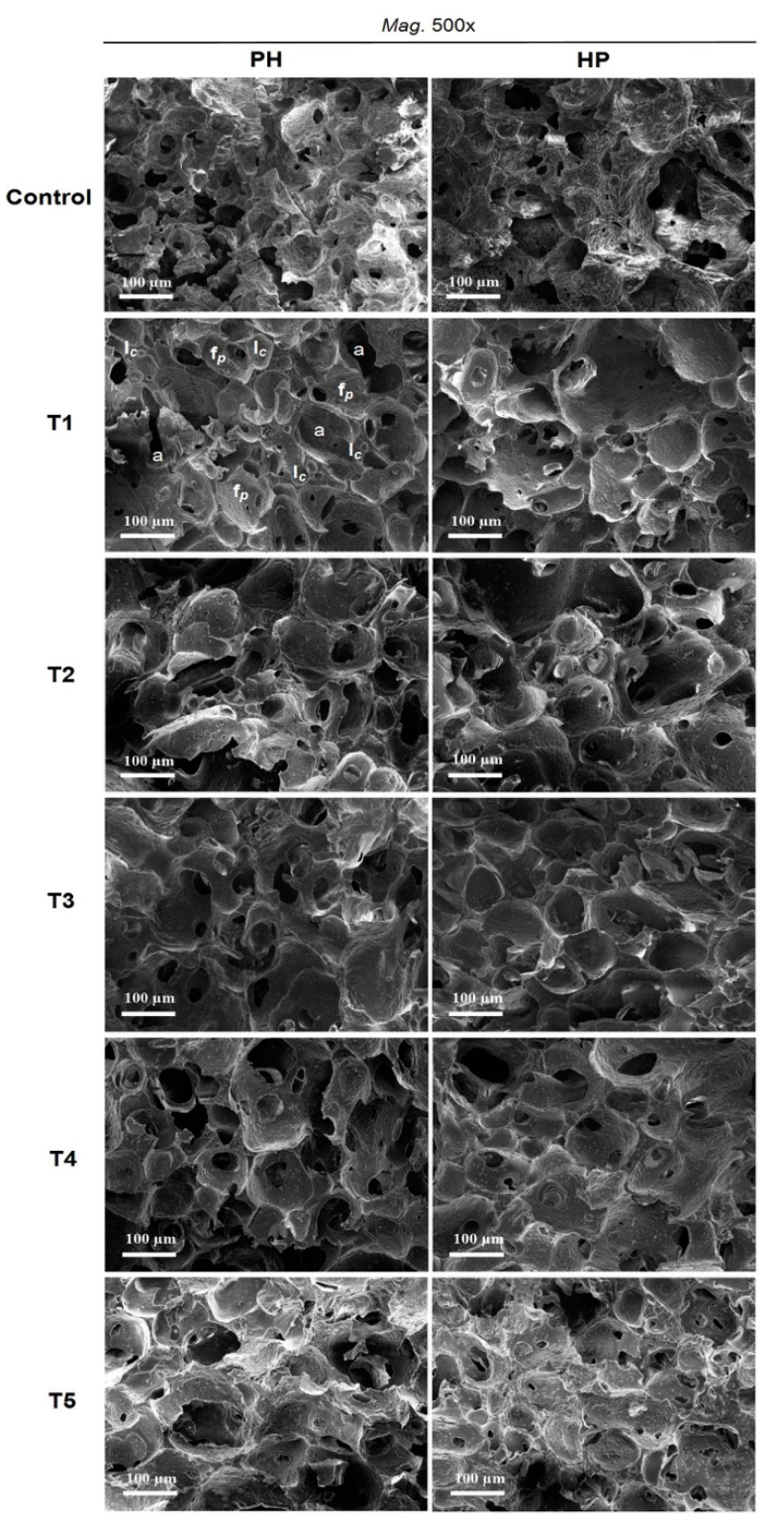
Microstructural properties of ice cream samples including MWPs at a magnification of 500×; *a* denotes integrated air bubbles, *I_c_* denotes ice crystals, and *f_p_* denotes fat globules and integrated particles of MWPs on the surface of ice cream. See Table 1 for the definitions of treatment abbreviations.

Microstructural images of the light ice cream treatments (T1–T5) and control sample made with the two process designs were taken at a magnification of 500× (Figure 5), followed by magnifications of 250× and 1000× (Figures S1 and S2). The control ice cream made with PH had more uniform and comparatively smaller pore sizes than did the treatments formulated with inulin, Simplesse, and eMWPs. HP resulted in a microstructure with more visible fat globules and aggregated protein particles compared to PH (Figure S2); the reason for that could be due to heat treatment after homogenization, resulting in a higher chance of forming larger aggregates. For both process designs, T1 had a relatively more compact microstructure and greater density of air bubbles in comparison with all other treatments, as shown in Figure S1. Several authors have established that using fat replacements in ice cream, including inulin and eMWPs, leads to the formation of a gel network through the interaction between the stabilizer–emulsifier and microparticulated protein aggregates, resulting in uniform microstructures [4,7,39]. T1 and T3, which contained inulin as a fat replacer, had relatively smoother surfaces compared to T2, T4, and T5 for both process designs. The only difference was that, in T5, there were a large number of aggregated protein particles and fat globules compared to T4; other microstructural properties, e.g., incorporated air bubbles and ice crystals, were similar. It has already been established that milk proteins help to enhance the microstructure of ice cream, including the foaming structure; stabilize the air bubble interface; ensure microstructural stability; and improve other physicochemical properties [14,40].

Microstructural properties are strongly related to the process steps, such as homogenization and pasteurization (Figure 5), and other physicochemical properties such as melting behavior, firmness, and overrun are strongly correlated with each other (Table S1). Homogenization of an ice cream mix affects the size distribution of fat globules, which can influence the aggregation of fat globules during the freezing process and enhance other physicochemical properties, such as overrun and melting behavior [7]. Temperature is the main factor that determines crystallization behavior in the ice cream manufacturing process, and it has significant effects on the microstructure, rheological behavior, and recrystallization during storage [22].

## 4. Conclusions

This study discovered that the manufacturing process of light ice cream (6% fat) is affected by the air incorporation, melting behavior, and viscosity of the ice cream, and when eMWPs are used, the microstructural properties are also affected. Light ice cream containing inulin (T1) had similar melting behavior and higher overrun, firmness, and viscosity compared to the control (12% fat). T2 had a faster melting behavior than did T4 and T5 in both process designs because of the longer rehydration time required for MWPs. Due to the positive effects on physicochemical properties (e.g., firmness and melting behavior), inulin and eMWPs can be used to enhance the structural quality of light ice cream. The microparticle size also influenced the texture of light ice cream; smaller particles (d_50_ < 3 µm) resulted in softer ice cream compared to larger particles (d_50_ > 5 µm). In contrast, in the case of air incorporation, the smaller particles of eMWPs showed higher overrun than did the larger particles. The strong correlation among physicochemical properties clarifies the effects of using eMWPs in light ice cream compared to full-fat ice cream. The microstructural and functional properties of eMWPs in both process designs are particularly important to understand the structural quality of ice cream. The rehydration of MWPs as well as different sources of microparticles and hydrocolloids need to be further investigated in relation to the thermomechanical process of light ice cream production.

## Figures and Tables

**Table 1:** Formulation of all treatments with and without microparticulated whey proteins.

Major Ingredients	Treatments ^a^					
	Control	T1	T2	T3	T4	T5
Fat ^f^ (%)	12	6	6	6	6	6
LSM ^b^ (g)	445	639	639	639	517	515
Cream (g)	390	190	190	190	177	177
SMP ^c^ (g)	54	–	–	–	10	10
Sugar (g)	105	105	105	105	105	105
eMWP ^d,f^ d_50_ < 3µm (g)	–	–	–	–	185	–
eMWP ^e,f^ d_50_ > 5 µm (g)	–	–	–	–	–	187
Inulin ^f^ (g)	–	60	–	20	–	–
Simplesse ^f^ (g)	–	–	60	40	–	–
Palsgaard (g)	3	3	3	3	3	3
Xanthan gum (g)	1	1	1	1	1	1
Vanilla flavour (g)	2	2	2	2	2	2

^a^: Formulations of all ice cream treatments are defined, including the control. ^b^: Liquid skimmed milk (LSM): used in all treatments, especially those that are reduced fat (T1–T5). ^c^: Skimmed milk powder (SMP): quantity differs to adjust the dry matter of all treatments. ^d^: Extruded microparticulated whey protein (eMWPs, d_50_ < 3 µm) quantity differs based on the dry matter content of the suspension. ^e^: eMWPs, d_50_ > 5 µm and the number of eMWPs are chosen on the basis of overall protein concentration (C_protein_). ^f^: These are considered factors, including T3 (a combination of simplesse and inulin). – Data is not available.

**Table 2:** Chemical composition of all treatments with and without microparticulated whey proteins, including the control sample.

Treatments ^4^	Components ^1^ (%)					
	Dry Matter		Fat		Protein	
	PH ^2^	HP ^3^	PH ^2^	HP ^3^	PH ^2^	HP ^3^
Control	35.57 ± 1.59 ^b^	35.91 ± 1.22 ^B^	12.56 ± 0.77 ^b^	13.04 ± 0.76 ^B^	6.04 ± 0.28 ^a^	6.15 ± 0.21 ^AB^
T1	31.76 ± 0.35 ^a^	32.44 ± 0.15 ^A^	6.80 ± 0.67 ^a^	7.09 ± 0.30 ^A^	5.07 ± 0.11 ^a^	5.09 ± 0.12 ^A^
T2	32.63 ± 0.76 ^a^	30.64 ± 0.29 ^A^	7.09 ± 0.30 ^a^	6.27 ± 0.51 ^A^	6.94 ± 0.66 ^ab^	6.43 ± 1.06 ^ABC^
T3	31.79 ± 1.63 ^a^	31.75 ± 1.68 ^A^	6.73 ± 0.24 ^a^	6.05 ± 0.57 ^A^	5.75 ± 0.46 ^a^	5.62 ± 0.51 ^AB^
T4	30.57 ± 1.97 ^a^	32.18 ± 2.01 ^A^	6.58 ± 0.86 ^a^	6.57 ± 0.85 ^A^	8.77 ± 0.97 ^b^	8.97 ± 0.88 ^C^
T5	32.91 ± 2.46 ^a^	30.79 ± 2.54 ^A^	6.71 ± 0.44 ^a^	6.48 ± 0.52 ^A^	7.55 ± 1.38 ^ab^	8.12 ± 1.40 ^BC^

^1^: Mean ± standard deviation of all ice cream treatments in percentage. ^2^: ^a,b^—denotes the level of significance at *p* < 0.05 within the process variation of PH; the same letter indicates that the treatments are not significantly different from each other. ^3^: ^A–C^—denotes the level of significance at *p* < 0.05 within the process technique HP; the same letter indicates that the treatments are not significantly different from each other. ^4^: See Table 1 for the definitions of treatment abbreviations (F1–F5).

**Table 3:** Color properties of all treatments with and without microparticulated whey proteins, including the control sample.

Treatments ^4^	Color properties ^1^							
	∆*E*		L*		a *		b *	
	PH ^2^	HP ^3^	PH ^2^	HP ^3^	PH ^2^	HP ^3^	PH ^2^	HP ^3^
Control	90.75 ± 0.49 ^a^	88.43 ± 1.51 ^A^	0.097 ± 0.09 ^a^	0.247 ± 0.12 ^A^	14.48 ± 0.47 ^a^	15.34 ± 0.84 ^A^	–	–
T1	87.99 ± 1.44 ^ab^	87.72 ± 1.86 ^A^	0.077 ± 0.07 ^a^	0.100 ± 0.07 ^A^	10.69 ± 0.36 ^d^	10.66 ± 0.47 ^C^	4.77 ± 1.05 ^a^	5.47 ± 0.94 ^A^
T2	84.43 ± 0.84 ^cd^	84.53 ± 1.68 ^AB^	0.197 ± 0.02 ^a^	0.153 ± 0.16 ^A^	13.38 ± 0.14 ^ab^	13.79 ± 0.79 ^AB^	6.46 ± 1.20 ^a^	4.65 ± 1.28 ^A^
T3	86.94 ± 0.84 ^bc^	86.27 ± 1.45 ^AB^	0.177 ± 0.10 ^a^	0.127 ± 0.03 ^A^	11.40 ± 0.65 ^cd^	11.68 ± 0.79 ^BC^	5.02 ± 0.03 ^a^	4.61 ± 1.23 ^A^
T4	84.55 ± 0.55 ^cd^	81.57 ± 1.20 ^B^	0.093 ± 0.04 ^a^	0.310 ± 0.20 ^A^	12.71 ± 0.36 ^bc^	14.01 ± 0.73 ^AB^	6.46 ± 0.75 ^a^	7.17 ± 2.17 ^A^
T5	83.81 ± 0.46 ^d^	82.84 ± 0.30 ^B^	0.150 ± 0.17 ^a^	0.183 ± 0.07 ^A^	14.01 ± 0.36 ^ab^	13.62 ± 0.47 ^AB^	7.00 ± 0.78 ^a^	5.88 ± 1.83 ^A^

^1^: Mean ± standard deviation of all ice cream treatments with the CIE L*a*b* color measurement system. ^2^: ^a–d^ denote the level of significance at *p* < 0.05 within the process variation of PH; the same letter indicates that the treatments are not significantly different from each other. ^3^: ^A–C^ denote the level of significance at *p* < 0.05 within HP; the same letter indicates that the treatments are not significantly different from each other. ^4^: See Table 1 for the definitions of treatment abbreviations (F1–F5). – Data is not available.

## Data Availability

Not applicable.

## Supplementary Materials

The following are available online at https://www.mdpi.com/article/10.3390/foods10061433/s1, Figure S1: Microstructural properties of ice cream samples including MWPs at a magnification of 250×; Figure S2: Microstructural properties of ice cream samples including MWPs at a magnification of 1000×; Table S1: Correlation and regression table of physicochemical properties of ice cream samples.
