# Supplementary material for: Microstructure and Physicochemical Properties of Light Ice Cream: Effects of Extruded Microparticulated Whey Proteins and Process Design
Source: Foods. 2021 Jun 21;10(6):1433. doi: 10.3390/foods10061433 (PMC8234353; doi:10.3390/foods10061433)
Supplement: Supplementary file 1 [file foods-10-01433-s001.zip › Fig. S1 Microstructure 250x.pdf]

Supplementary information

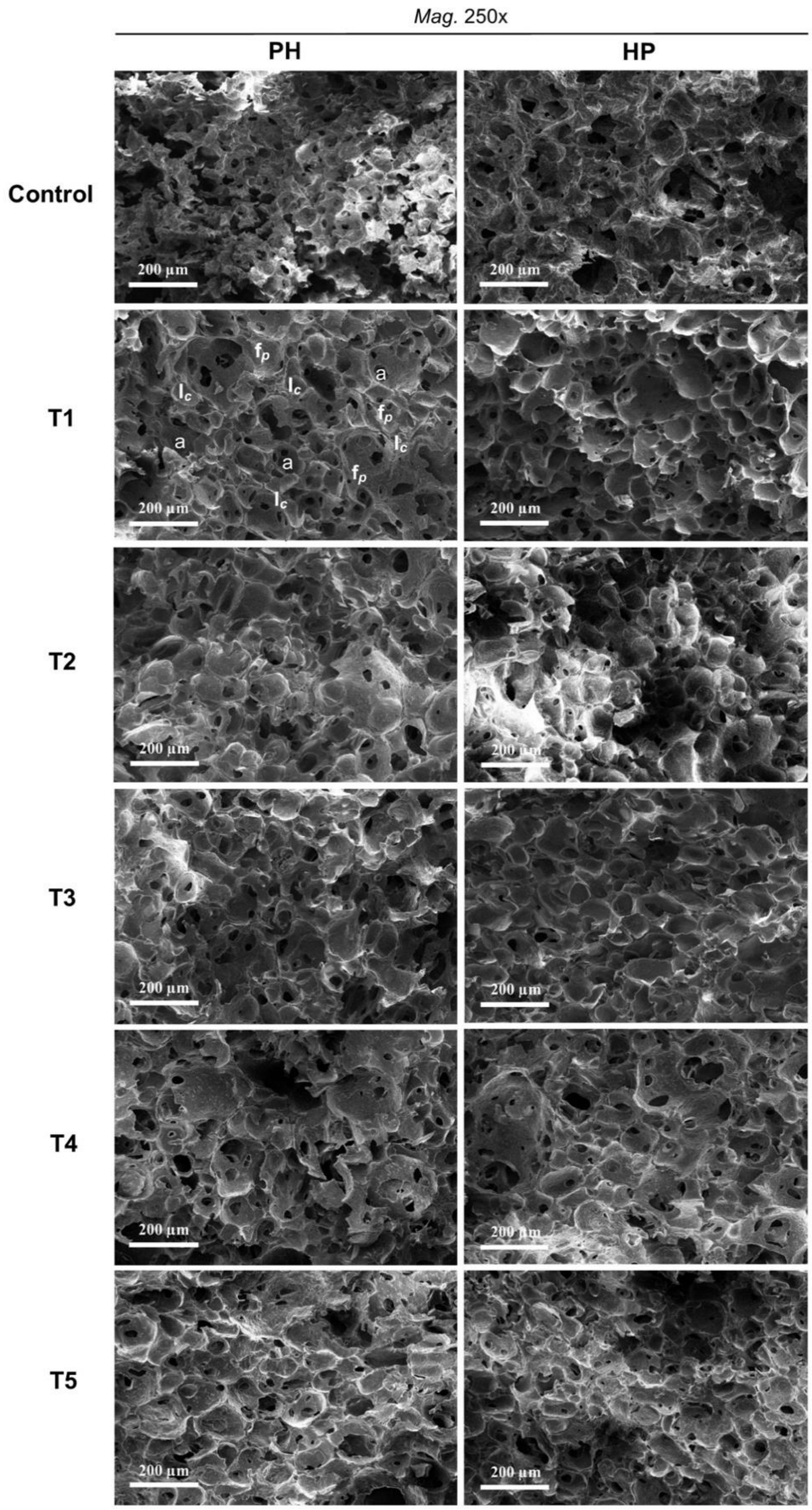

a – integrated air bubbles.
I_c – ice crystals.
f_p – fat globules and integrated particles of MWPs on the ice cream surface.

Figure S1. Microstructural properties of ice cream samples including MWPs at a magnification of 250x.

See **Table 1** for the definition of treatment abbreviations.

Key finding:
Relatively more compact microstructure of ice cream treatments and the density of air bubbles observed in both cases PH and HP.
