# Supplementary material for: Microstructure and Physicochemical Properties of Light Ice Cream: Effects of Extruded Microparticulated Whey Proteins and Process Design
Source: Foods. 2021 Jun 21;10(6):1433. doi: 10.3390/foods10061433 (PMC8234353; doi:10.3390/foods10061433)
Supplement: Supplementary file 1 [file foods-10-01433-s001.zip › Fig. S2 Microstructure 1000x.pdf]

Supplementary information

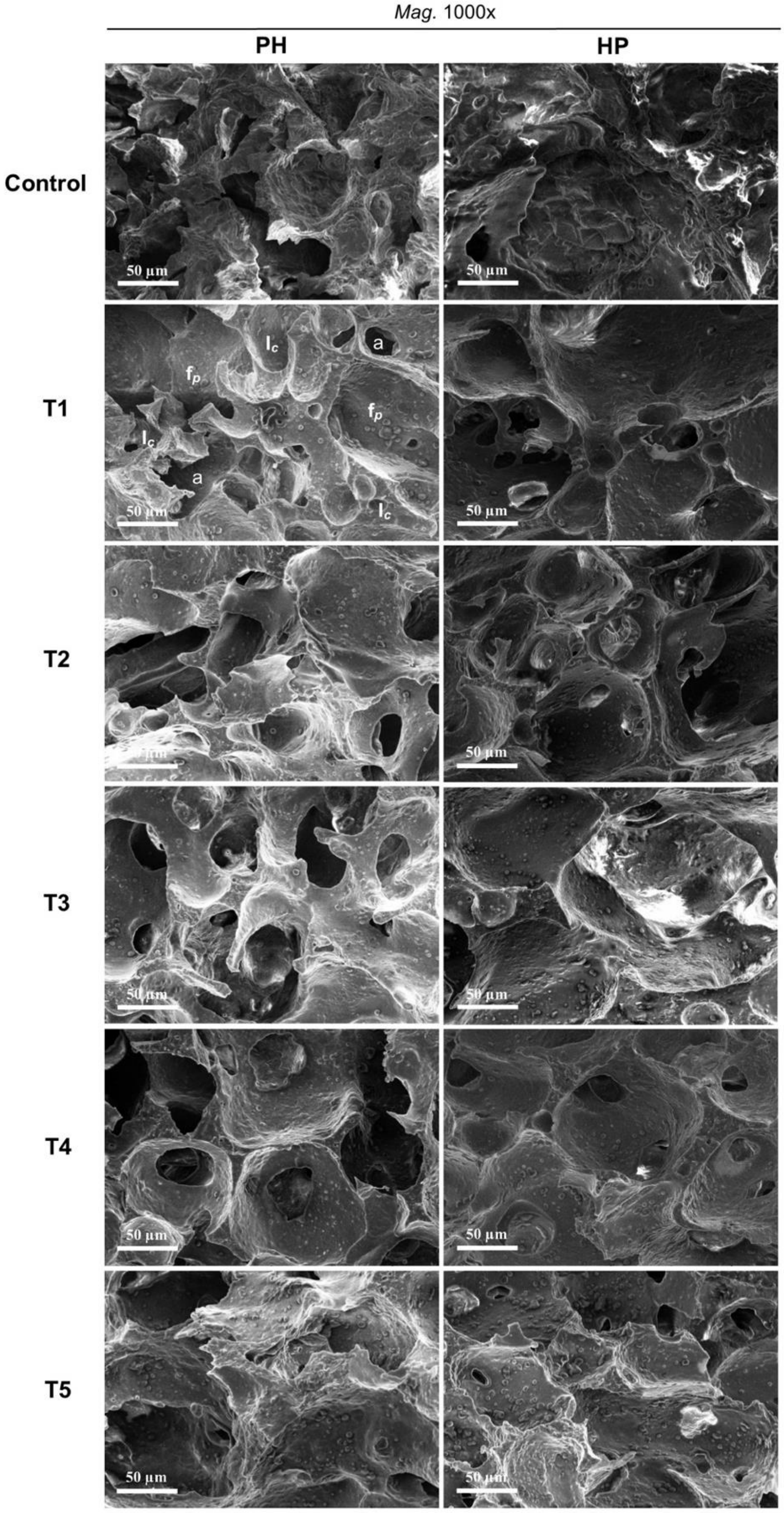

Figure S2. Microstructural properties of ice cream samples including MWPs at a magnification of 1000x.

See Table 1 for the definition of treatment abbreviations.

Key finding:
Clear visibility of fat globules and aggregated protein particles in ice cream treatments subjected to HP than PH.

a – integrated air bubbles.
I_c – ice crystals.
f_p – fat globules and integrated particles of MWPs on the ice cream surface.
