# Supplementary material for: Microstructure and Physicochemical Properties of Light Ice Cream: Effects of Extruded Microparticulated Whey Proteins and Process Design
Source: Foods. 2021 Jun 21;10(6):1433. doi: 10.3390/foods10061433 (PMC8234353; doi:10.3390/foods10061433)
Supplement: Supplementary file 1 [file foods-10-01433-s001.zip › Table S1 Correlation Table.pdf]

Supplementary information

Table S1 Correlation and regression table of physicochemical properties of ice creams.

	PH_Fir (1)	HP_Fir (2)	PH_Mel (3)	HP_Mel (4)	PH_Ove (5)	HP_Ove (6)	PH_Vis (7)	HP_Vis (8)	PH_DM (9)	HP_DM (10)	PH_Pro (11)	HP_Pro (12)	PH_Fat (13)	HP_Fat (14)
PH_Firmness (1)	1													
HP_Firmness (2)	0.94***	1												
PH_Melting (3)	0.39	0.45	1											
HP_Melting (4)	0.23	0.22	0.29	1										
PH_Overrun (5)	0.03	-0.07	0.32	0.09	1									
HP_Overrun (6)	0.02	0.04	0.32	0.20	-0.17	1								
PH_Viscosity (7)	-0.42	-0.58*	-0.68**	-0.23	-0.05	-0.27	1							
HP_Viscosity (8)	0.38	0.35	-0.15	-0.39	-0.02	-0.50*	0.28	1						
PH_Dry Matter (9)	-0.45	-0.47*	-0.28	-0.12	-0.04	-0.26	0.08	-0.19	1					
HP_Dry Matter (10)	-0.58*	-0.50*	-0.45	-0.52*	-0.18	-0.04	0.40	0.04	0.22	1				
PH_Protein (11)	0.49*	0.55*	0.74**	0.44	0.18	0.44	-0.63*	-0.03	-0.16	-0.62*	1			
HP_Protein (12)	0.60*	0.71*	0.49*	0.21	0.04	0.38	-0.62*	0.18	-0.45	-0.37	0.69**	1		
PH_Fat (13)	-0.58*	-0.46	-0.39	-0.41	-0.21	0.11	0.01	-0.18	0.50*	0.43	-0.19	-0.19	1	
HP_Fat (14)	-0.55*	-0.43	-0.31	-0.64*	-0.15	0.01	0.02	0.01	0.45	0.50*	-0.22	-0.18	0.94***	1

PH – Pasteurization with subsequent homogenization.

HP – Homogenization with subsequent pasteurization.

* Significant level at $p < 0.05$

** Significant level at $p < 0.01$

*** Significant level at $p < 0.001$
